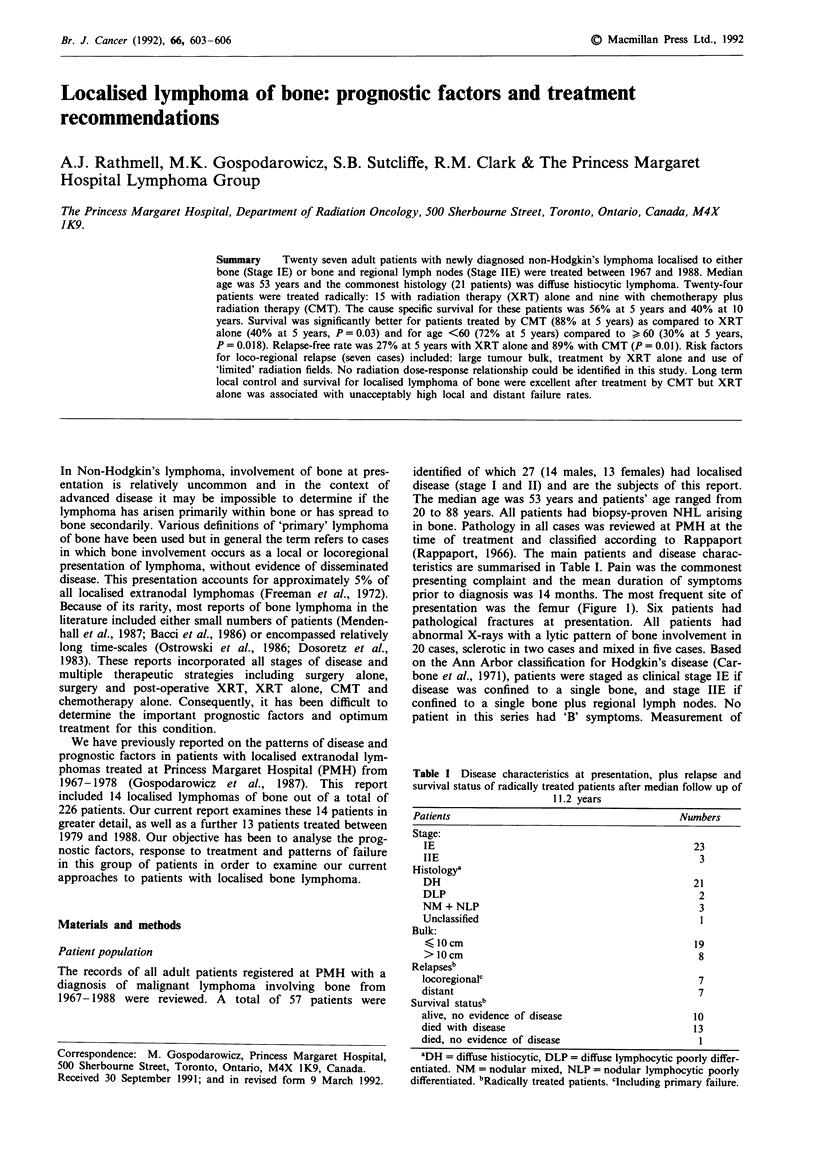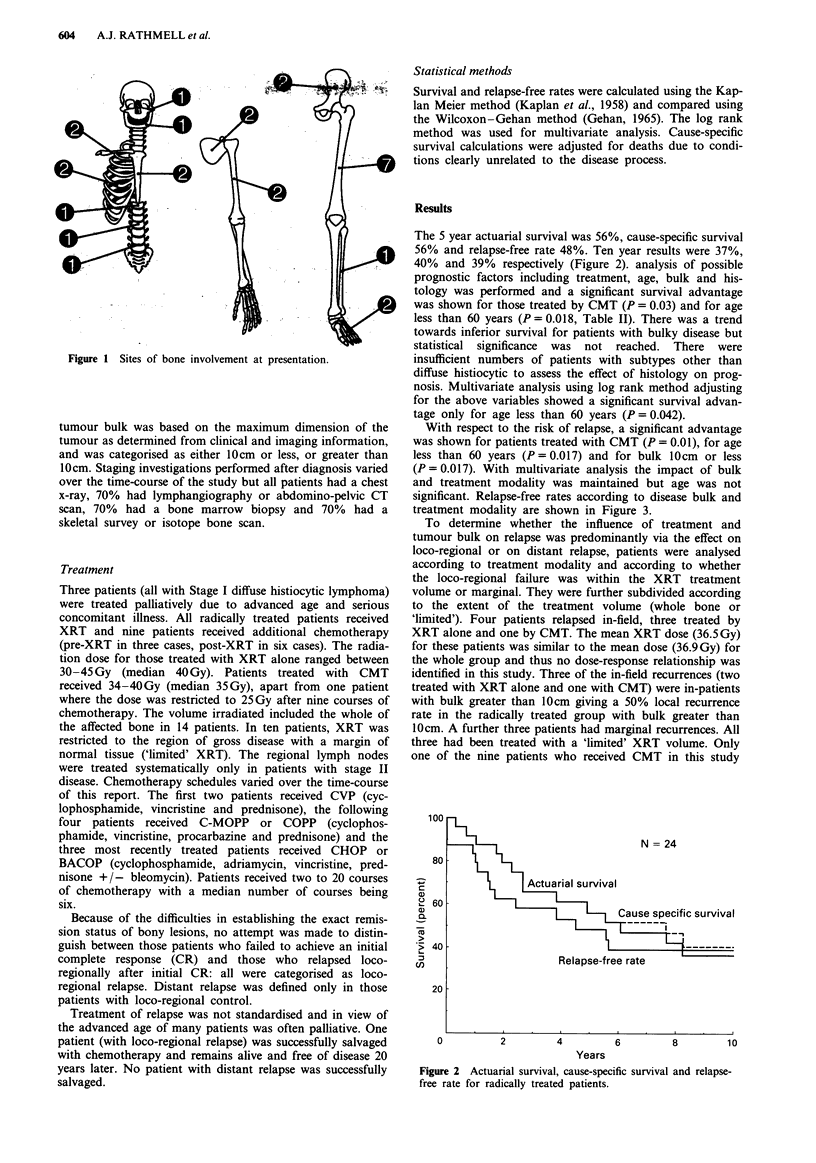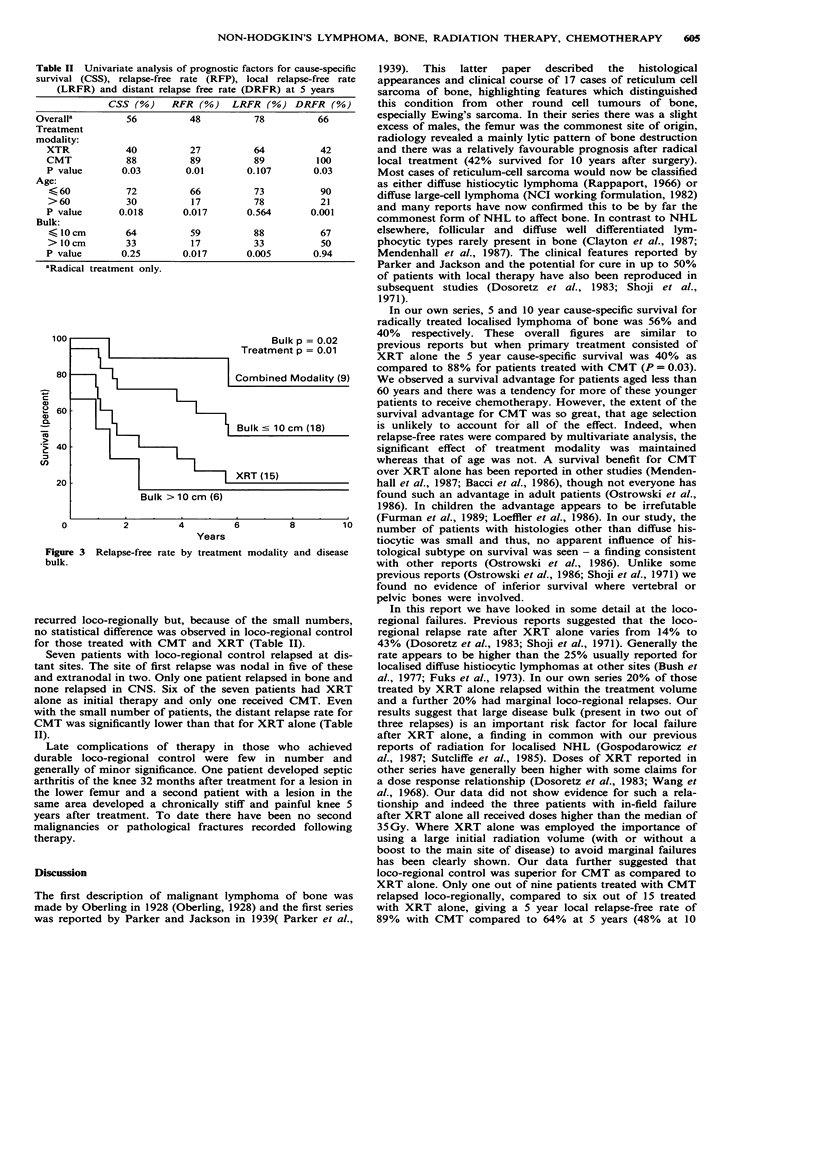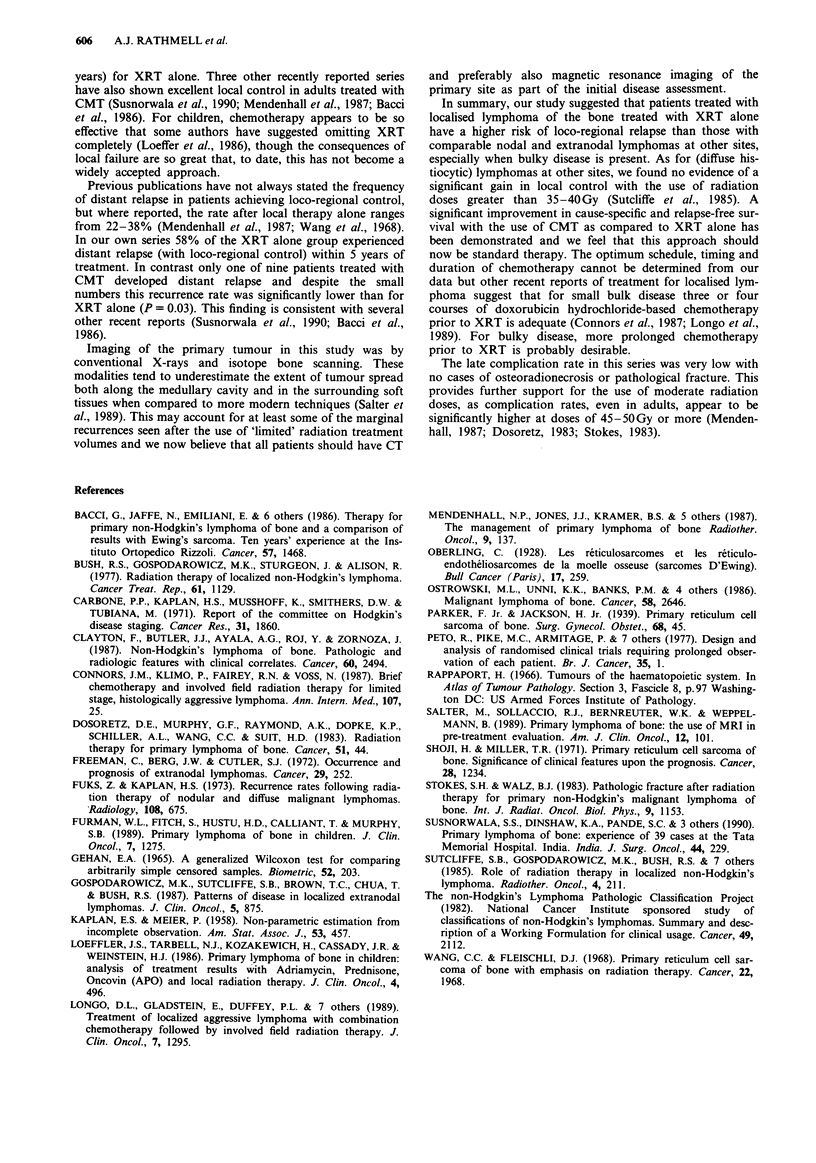# Localised lymphoma of bone: prognostic factors and treatment recommendations. The Princess Margaret Hospital Lymphoma Group.

**DOI:** 10.1038/bjc.1992.322

**Published:** 1992-09

**Authors:** A. J. Rathmell, M. K. Gospodarowicz, S. B. Sutcliffe, R. M. Clark

**Affiliations:** Princess Margaret Hospital, Department of Radiation Oncology, Toronto, Ontario, Canada.

## Abstract

Twenty seven adult patients with newly diagnosed non-Hodgkin's lymphoma localised to either bone (Stage IE) or bone and regional lymph nodes (Stage IIE) were treated between 1967 and 1988. Median age was 53 years and the commonest histology (21 patients) was diffuse histiocytic lymphoma. Twenty-four patients were treated radically: 15 with radiation therapy (XRT) alone and nine with chemotherapy plus radiation therapy (CMT). The cause specific survival for these patients was 56% at 5 years and 40% at 10 years. Survival was significantly better for patients treated by CMT (88% at 5 years) as compared to XRT alone (40% at 5 years, P = 0.03) and for age less than 60 (72% at 5 years) compared to greater than or equal to 60 (30% at 5 years, P = 0.018). Relapse-free rate was 27% at 5 years with XRT alone and 89% with CMT (P = 0.01). Risk factors for loco-regional relapse (seven cases) included: large tumour bulk, treatment by XRT alone and use of 'limited' radiation fields. No radiation dose-response relationship could be identified in this study. Long term local control and survival for localised lymphoma of bone were excellent after treatment by CMT but XRT alone was associated with unacceptably high local and distant failure rates.


Br. J. Cancer (1992), 66, 603-606 © Macmillan Press Ltd., 1992

### Localised lymphoma of bone: prognostic factors and treatment recommendations

A.J. Rathmell, M.K. Gospodarowicz, S.B. Sutcliffe, R.M. Clark & The Princess Margaret Hospital Lymphoma Group

*The Princess Margaret Hospital, Department of Radiation Oncology, 500 Sherbourne Street, Toronto, Ontario, Canada, M4X 1K9.*

**Summary** Twenty seven adult patients with newly diagnosed non-Hodgkin's lymphoma localised to either bone (Stage IE) or bone and regional lymph nodes (Stage IIE) were treated between 1967 and 1988. Median age was 53 years and the commonest histology (21 patients) was diffuse histiocytic lymphoma. Twenty-four patients were treated radically: 15 with radiation therapy (XRT) alone and nine with chemotherapy plus radiation therapy (CMT). The cause specific survival for these patients was 56% at 5 years and 40% at 10 years. Survival was significantly better for patients treated by CMT (88% at 5 years) as compared to XRT alone (40% at 5 years, $P = 0.03$) and for age <60 (72% at 5 years) compared to $\geqslant 60$ (30% at 5 years, $P = 0.018$). Relapse-free rate was 27% at 5 years with XRT alone and 89% with CMT ($P = 0.01$). Risk factors for loco-regional relapse (seven cases) included: large tumour bulk, treatment by XRT alone and use of 'limited' radiation fields. No radiation dose-response relationship could be identified in this study. Long term local control and survival for localised lymphoma of bone were excellent after treatment by CMT but XRT alone was associated with unacceptably high local and distant failure rates.

In Non-Hodgkin's lymphoma, involvement of bone at presentation is relatively uncommon and in the context of advanced disease it may be impossible to determine if the lymphoma has arisen primarily within bone or has spread to bone secondarily. Various definitions of 'primary' lymphoma of bone have been used but in general the term refers to cases in which bone involvement occurs as a local or locoregional presentation of lymphoma, without evidence of disseminated disease. This presentation accounts for approximately 5% of all localised extranodal lymphomas (Freeman *et al.*, 1972). Because of its rarity, most reports of bone lymphoma in the literature included either small numbers of patients (Mendenhall *et al.*, 1987; Bacci *et al.*, 1986) or encompassed relatively long time-scales (Ostrowski *et al.*, 1986; Dosoretz *et al.*, 1983). These reports incorporated all stages of disease and multiple therapeutic strategies including surgery alone, surgery and post-operative XRT, XRT alone, CMT and chemotherapy alone. Consequently, it has been difficult to determine the important prognostic factors and optimum treatment for this condition.

We have previously reported on the patterns of disease and prognostic factors in patients with localised extranodal lymphomas treated at Princess Margaret Hospital (PMH) from 1967–1978 (Gospodarowicz *et al.*, 1987). This report included 14 localised lymphomas of bone out of a total of 226 patients. Our current report examines these 14 patients in greater detail, as well as a further 13 patients treated between 1979 and 1988. Our objective has been to analyse the prognostic factors, response to treatment and patterns of failure in this group of patients in order to examine our current approaches to patients with localised bone lymphoma.

### Materials and methods

### *Patient population*

The records of all adult patients registered at PMH with a diagnosis of malignant lymphoma involving bone from 1967–1988 were reviewed. A total of 57 patients were identified of which 27 (14 males, 13 females) had localised disease (stage I and II) and are the subjects of this report. The median age was 53 years and patients' age ranged from 20 to 88 years. All patients had biopsy-proven NHL arising in bone. Pathology in all cases was reviewed at PMH at the time of treatment and classified according to Rappaport (Rappaport, 1966). The main patients and disease characteristics are summarised in Table I. Pain was the commonest presenting complaint and the mean duration of symptoms prior to diagnosis was 14 months. The most frequent site of presentation was the femur (Figure 1). Six patients had pathological fractures at presentation. All patients had abnormal X-rays with a lytic pattern of bone involvement in 20 cases, sclerotic in two cases and mixed in five cases. Based on the Ann Arbor classification for Hodgkin's disease (Carbone *et al.*, 1971), patients were staged as clinical stage IE if disease was confined to a single bone, and stage IIE if confined to a single bone plus regional lymph nodes. No patient in this series had 'B' symptoms. Measurement of

Correspondence: M. Gospodarowicz, Princess Margaret Hospital, 500 Sherbourne Street, Toronto, Ontario, M4X 1K9, Canada.
Received 30 September 1991; and in revised form 9 March 1992.

**Table I** Disease characteristics at presentation, plus relapse and survival status of radically treated patients after median follow up of 11.2 years

| *Patients* | *Numbers* |
|---|---|
| Stage: | |
| IE | 23 |
| IIE | 3 |
| Histology[a] | |
| DH | 21 |
| DLP | 2 |
| NM + NLP | 3 |
| Unclassified | 1 |
| Bulk: | |
| $\leqslant 10$ cm | 19 |
| $> 10$ cm | 8 |
| Relapses[b] | |
| locoregional[c] | 7 |
| distant | 7 |
| Survival status[b] | |
| alive, no evidence of disease | 10 |
| died with disease | 13 |
| died, no evidence of disease | 1 |

[a]DH = diffuse histiocytic, DLP = diffuse lymphocytic poorly differentiated. NM = nodular mixed, NLP = nodular lymphocytic poorly differentiated. [b]Radically treated patients. [c]Including primary failure.

**Figure 1** Sites of bone involvement at presentation.

tumour bulk was based on the maximum dimension of the tumour as determined from clinical and imaging information, and was categorised as either 10cm or less, or greater than 10cm. Staging investigations performed after diagnosis varied over the time-course of the study but all patients had a chest x-ray, 70% had lymphangiography or abdomino-pelvic CT scan, 70% had a bone marrow biopsy and 70% had a skeletal survey or isotope bone scan.

### *Treatment*

Three patients (all with Stage I diffuse histiocytic lymphoma) were treated palliatively due to advanced age and serious concomitant illness. All radically treated patients received XRT and nine patients received additional chemotherapy (pre-XRT in three cases, post-XRT in six cases). The radiation dose for those treated with XRT alone ranged between 30–45Gy (median 40Gy). Patients treated with CMT received 34–40Gy (median 35Gy), apart from one patient where the dose was restricted to 25Gy after nine courses of chemotherapy. The volume irradiated included the whole of the affected bone in 14 patients. In ten patients, XRT was restricted to the region of gross disease with a margin of normal tissue ('limited' XRT). The regional lymph nodes were treated systematically only in patients with stage II disease. Chemotherapy schedules varied over the time-course of this report. The first two patients received CVP (cyclophosphamide, vincristine and prednisone), the following four patients received C-MOPP or COPP (cyclophosphamide, vincristine, procarbazine and prednisone) and the three most recently treated patients received CHOP or BACOP (cyclophosphamide, adriamycin, vincristine, prednisone +/− bleomycin). Patients received two to 20 courses of chemotherapy with a median number of courses being six.

Because of the difficulties in establishing the exact remission status of bony lesions, no attempt was made to distinguish between those patients who failed to achieve an initial complete response (CR) and those who relapsed loco-regionally after initial CR: all were categorised as loco-regional relapse. Distant relapse was defined only in those patients with loco-regional control.

Treatment of relapse was not standardised and in view of the advanced age of many patients was often palliative. One patient (with loco-regional relapse) was successfully salvaged with chemotherapy and remains alive and free of disease 20 years later. No patient with distant relapse was successfully salvaged.

### *Statistical methods*

Survival and relapse-free rates were calculated using the Kaplan Meier method (Kaplan *et al.*, 1958) and compared using the Wilcoxon–Gehan method (Gehan, 1965). The log rank method was used for multivariate analysis. Cause-specific survival calculations were adjusted for deaths due to conditions clearly unrelated to the disease process.

### Results

The 5 year actuarial survival was 56%, cause-specific survival 56% and relapse-free rate 48%. Ten year results were 37%, 40% and 39% respectively (Figure 2). analysis of possible prognostic factors including treatment, age, bulk and histology was performed and a significant survival advantage was shown for those treated by CMT ($P = 0.03$) and for age less than 60 years ($P = 0.018$, Table II). There was a trend towards inferior survival for patients with bulky disease but statistical significance was not reached. There were insufficient numbers of patients with subtypes other than diffuse histiocytic to assess the effect of histology on prognosis. Multivariate analysis using log rank method adjusting for the above variables showed a significant survival advantage only for age less than 60 years ($P = 0.042$).

With respect to the risk of relapse, a significant advantage was shown for patients treated with CMT ($P = 0.01$), for age less than 60 years ($P = 0.017$) and for bulk 10cm or less ($P = 0.017$). With multivariate analysis the impact of bulk and treatment modality was maintained but age was not significant. Relapse-free rates according to disease bulk and treatment modality are shown in Figure 3.

To determine whether the influence of treatment and tumour bulk on relapse was predominantly via the effect on loco-regional or on distant relapse, patients were analysed according to treatment modality and according to whether the loco-regional failure was within the XRT treatment volume or marginal. They were further subdivided according to the extent of the treatment volume (whole bone or 'limited'). Four patients relapsed in-field, three treated by XRT alone and one by CMT. The mean XRT dose (36.5Gy) for these patients was similar to the mean dose (36.9Gy) for the whole group and thus no dose-response relationship was identified in this study. Three of the in-field recurrences (two treated with XRT alone and one with CMT) were in-patients with bulk greater than 10cm giving a 50% local recurrence rate in the radically treated group with bulk greater than 10cm. A further three patients had marginal recurrences. All three had been treated with a 'limited' XRT volume. Only one of the nine patients who received CMT in this study

**Figure 2** Actuarial survival, cause-specific survival and relapse-free rate for radically treated patients.

**Table II** Univariate analysis of prognostic factors for cause-specific survival (CSS), relapse-free rate (RFP), local relapse-free rate (LRFR) and distant relapse free rate (DRFR) at 5 years

| | CSS (%) | RFR (%) | LRFR (%) | DRFR (%) |
|---|---|---|---|---|
| Overall[a] | 56 | 48 | 78 | 66 |
| Treatment modality: | | | | |
| XTR | 40 | 27 | 64 | 42 |
| CMT | 88 | 89 | 89 | 100 |
| P value | 0.03 | 0.01 | 0.107 | 0.03 |
| Age: | | | | |
| ⩽60 | 72 | 66 | 73 | 90 |
| >60 | 30 | 17 | 78 | 21 |
| P value | 0.018 | 0.017 | 0.564 | 0.001 |
| Bulk: | | | | |
| ⩽10 cm | 64 | 59 | 88 | 67 |
| >10 cm | 33 | 17 | 33 | 50 |
| P value | 0.25 | 0.017 | 0.005 | 0.94 |

[a]Radical treatment only.

**Figure 3** Relapse-free rate by treatment modality and disease bulk.

recurred loco-regionally but, because of the small numbers, no statistical difference was observed in loco-regional control for those treated with CMT and XRT (Table II).

Seven patients with loco-regional control relapsed at distant sites. The site of first relapse was nodal in five of these and extranodal in two. Only one patient relapsed in bone and none relapsed in CNS. Six of the seven patients had XRT alone as initial therapy and only one received CMT. Even with the small number of patients, the distant relapse rate for CMT was significantly lower than that for XRT alone (Table II).

Late complications of therapy in those who achieved durable loco-regional control were few in number and generally of minor significance. One patient developed septic arthritis of the knee 32 months after treatment for a lesion in the lower femur and a second patient with a lesion in the same area developed a chronically stiff and painful knee 5 years after treatment. To date there have been no second malignancies or pathological fractures recorded following therapy.

### Discussion

The first description of malignant lymphoma of bone was made by Oberling in 1928 (Oberling, 1928) and the first series was reported by Parker and Jackson in 1939( Parker *et al.*, 1939). This latter paper described the histological appearances and clinical course of 17 cases of reticulum cell sarcoma of bone, highlighting features which distinguished this condition from other round cell tumours of bone, especially Ewing's sarcoma. In their series there was a slight excess of males, the femur was the commonest site of origin, radiology revealed a mainly lytic pattern of bone destruction and there was a relatively favourable prognosis after radical local treatment (42% survived for 10 years after surgery). Most cases of reticulum-cell sarcoma would now be classified as either diffuse histiocytic lymphoma (Rappaport, 1966) or diffuse large-cell lymphoma (NCI working formulation, 1982) and many reports have now confirmed this to be by far the commonest form of NHL to affect bone. In contrast to NHL elsewhere, follicular and diffuse well differentiated lymphocytic types rarely present in bone (Clayton *et al.*, 1987; Mendenhall *et al.*, 1987). The clinical features reported by Parker and Jackson and the potential for cure in up to 50% of patients with local therapy have also been reproduced in subsequent studies (Dosoretz *et al.*, 1983; Shoji *et al.*, 1971).

In our own series, 5 and 10 year cause-specific survival for radically treated localised lymphoma of bone was 56% and 40% respectively. These overall figures are similar to previous reports but when primary treatment consisted of XRT alone the 5 year cause-specific survival was 40% as compared to 88% for patients treated with CMT ($P = 0.03$). We observed a survival advantage for patients aged less than 60 years and there was a tendency for more of these younger patients to receive chemotherapy. However, the extent of the survival advantage for CMT was so great, that age selection is unlikely to account for all of the effect. Indeed, when relapse-free rates were compared by multivariate analysis, the significant effect of treatment modality was maintained whereas that of age was not. A survival benefit for CMT over XRT alone has been reported in other studies (Mendenhall *et al.*, 1987; Bacci *et al.*, 1986), though not everyone has found such an advantage in adult patients (Ostrowski *et al.*, 1986). In children the advantage appears to be irrefutable (Furman *et al.*, 1989; Loeffler *et al.*, 1986). In our study, the number of patients with histologies other than diffuse histiocytic was small and thus, no apparent influence of histological subtype on survival was seen – a finding consistent with other reports (Ostrowski *et al.*, 1986). Unlike some previous reports (Ostrowski *et al.*, 1986; Shoji *et al.*, 1971) we found no evidence of inferior survival where vertebral or pelvic bones were involved.

In this report we have looked in some detail at the loco-regional failures. Previous reports suggested that the loco-regional relapse rate after XRT alone varies from 14% to 43% (Dosoretz *et al.*, 1983; Shoji *et al.*, 1971). Generally the rate appears to be higher than the 25% usually reported for localised diffuse histiocytic lymphomas at other sites (Bush *et al.*, 1977; Fuks *et al.*, 1973). In our own series 20% of those treated by XRT alone relapsed within the treatment volume and a further 20% had marginal loco-regional relapses. Our results suggest that large disease bulk (present in two out of three relapses) is an important risk factor for local failure after XRT alone, a finding in common with our previous reports of radiation for localised NHL (Gospodarowicz *et al.*, 1987; Sutcliffe *et al.*, 1985). Doses of XRT reported in other series have generally been higher with some claims for a dose response relationship (Dosoretz *et al.*, 1983; Wang *et al.*, 1968). Our data did not show evidence for such a relationship and indeed the three patients with in-field failure after XRT alone all received doses higher than the median of 35Gy. Where XRT alone was employed the importance of using a large initial radiation volume (with or without a boost to the main site of disease) to avoid marginal failures has been clearly shown. Our data further suggested that loco-regional control was superior for CMT as compared to XRT alone. Only one out of nine patients treated with CMT relapsed loco-regionally, compared to six out of 15 treated with XRT alone, giving a 5 year local relapse-free rate of 89% with CMT compared to 64% at 5 years (48% at 10

years) for XRT alone. Three other recently reported series have also shown excellent local control in adults treated with CMT (Susnorwala *et al.*, 1990; Mendenhall *et al.*, 1987; Bacci *et al.*, 1986). For children, chemotherapy appears to be so effective that some authors have suggested omitting XRT completely (Loeffer *et al.*, 1986), though the consequences of local failure are so great that, to date, this has not become a widely accepted approach.

Previous publications have not always stated the frequency of distant relapse in patients achieving loco-regional control, but where reported, the rate after local therapy alone ranges from 22–38% (Mendenhall *et al.*, 1987; Wang *et al.*, 1968). In our own series 58% of the XRT alone group experienced distant relapse (with loco-regional control) within 5 years of treatment. In contrast only one of nine patients treated with CMT developed distant relapse and despite the small numbers this recurrence rate was significantly lower than for XRT alone ($P = 0.03$). This finding is consistent with several other recent reports (Susnorwala *et al.*, 1990; Bacci *et al.*, 1986).

Imaging of the primary tumour in this study was by conventional X-rays and isotope bone scanning. These modalities tend to underestimate the extent of tumour spread both along the medullary cavity and in the surrounding soft tissues when compared to more modern techniques (Salter *et al.*, 1989). This may account for at least some of the marginal recurrences seen after the use of 'limited' radiation treatment volumes and we now believe that all patients should have CT and preferably also magnetic resonance imaging of the primary site as part of the initial disease assessment.

In summary, our study suggested that patients treated with localised lymphoma of the bone treated with XRT alone have a higher risk of loco-regional relapse than those with comparable nodal and extranodal lymphomas at other sites, especially when bulky disease is present. As for (diffuse histiocytic) lymphomas at other sites, we found no evidence of a significant gain in local control with the use of radiation doses greater than 35–40 Gy (Sutcliffe *et al.*, 1985). A significant improvement in cause-specific and relapse-free survival with the use of CMT as compared to XRT alone has been demonstrated and we feel that this approach should now be standard therapy. The optimum schedule, timing and duration of chemotherapy cannot be determined from our data but other recent reports of treatment for localised lymphoma suggest that for small bulk disease three or four courses of doxorubicin hydrochloride-based chemotherapy prior to XRT is adequate (Connors *et al.*, 1987; Longo *et al.*, 1989). For bulky disease, more prolonged chemotherapy prior to XRT is probably desirable.

The late complication rate in this series was very low with no cases of osteoradionecrosis or pathological fracture. This provides further support for the use of moderate radiation doses, as complication rates, even in adults, appear to be significantly higher at doses of 45–50 Gy or more (Mendenhall, 1987; Dosoretz, 1983; Stokes, 1983).

### References

BACCI, G., JAFFE, N., EMILIANI, E. & 6 others (1986). Therapy for primary non-Hodgkin's lymphoma of bone and a comparison of results with Ewing's sarcoma. Ten years' experience at the Instituto Ortopedico Rizzoli. *Cancer*, **57**, 1468.

BUSH, R.S., GOSPODAROWICZ, M.K., STURGEON, J. & ALISON, R. (1977). Radiation therapy of localized non-Hodgkin's lymphoma. *Cancer Treat. Rep.*, **61**, 1129.

CARBONE, P.P., KAPLAN, H.S., MUSSHOFF, K., SMITHERS, D.W. & TUBIANA, M. (1971). Report of the committee on Hodgkin's disease staging. *Cancer Res.*, **31**, 1860.

CLAYTON, F., BUTLER, J.J., AYALA, A.G., ROJ, Y. & ZORNOZA, J. (1987). Non-Hodgkin's lymphoma of bone. Pathologic and radiologic features with clinical correlates. *Cancer*, **60**, 2494.

CONNORS, J.M., KLIMO, P., FAIREY, R.N. & VOSS, N. (1987). Brief chemotherapy and involved field radiation therapy for limited stage, histologically aggressive lymphoma. *Ann. Intern. Med.*, **107**, 25.

DOSORETZ, D.E., MURPHY, G.F., RAYMOND, A.K., DOPKE, K.P., SCHILLER, A.L., WANG, C.C. & SUIT, H.D. (1983). Radiation therapy for primary lymphoma of bone. *Cancer*, **51**, 44.

FREEMAN, C., BERG, J.W. & CUTLER, S.J. (1972). Occurrence and prognosis of extranodal lymphomas. *Cancer*, **29**, 252.

FUKS, Z. & KAPLAN, H.S. (1973). Recurrence rates following radiation therapy of nodular and diffuse malignant lymphomas. *Radiology*, **108**, 675.

FURMAN, W.L., FITCH, S., HUSTU, H.D., CALLIANT, T. & MURPHY, S.B. (1989). Primary lymphoma of bone in children. *J. Clin. Oncol.*, **7**, 1275.

GEHAN, E.A. (1965). A generalized Wilcoxon test for comparing arbitrarily simple censored samples. *Biometric*, **52**, 203.

GOSPODAROWICZ, M.K., SUTCLIFFE, S.B., BROWN, T.C., CHUA, T. & BUSH, R.S. (1987). Patterns of disease in localized extranodal lymphomas. *J. Clin. Oncol.*, **5**, 875.

KAPLAN, E.S. & MEIER, P. (1958). Non-parametric estimation from incomplete observation. *Am. Stat. Assoc. J.*, **53**, 457.

LOEFFLER, J.S., TARBELL, N.J., KOZAKEWICH, H., CASSADY, J.R. & WEINSTEIN, H.J. (1986). Primary lymphoma of bone in children: analysis of treatment results with Adriamycin, Prednisone, Oncovin (APO) and local radiation therapy. *J. Clin. Oncol.*, **4**, 496.

LONGO, D.L., GLADSTEIN, E., DUFFEY, P.L. & 7 others (1989). Treatment of localized aggressive lymphoma with combination chemotherapy followed by involved field radiation therapy. *J. Clin. Oncol.*, **7**, 1295.

MENDENHALL, N.P., JONES, J.J., KRAMER, B.S. & 5 others (1987). The management of primary lymphoma of bone *Radiother. Oncol.*, **9**, 137.

OBERLING, C. (1928). Les réticulosarcomes et les réticulo-endothéliosarcomes de la moelle osseuse (sarcomes D'Ewing). *Bull Cancer (Paris)*, **17**, 259.

OSTROWSKI, M.L., UNNI, K.K., BANKS, P.M. & 4 others (1986). Malignant lymphoma of bone. *Cancer*, **58**, 2646.

PARKER, F. Jr. & JACKSON, H. Jr. (1939). Primary reticulum cell sarcoma of bone. *Surg. Gynecol. Obstet.*, **68**, 45.

PETO, R., PIKE, M.C., ARMITAGE, P. & 7 others (1977). Design and analysis of randomised clinical trials requiring prolonged observation of each patient. *Br. J. Cancer*, **35**, 1.

RAPPAPORT, H. (1966). Tumours of the haematopoietic system. In *Atlas of Tumour Pathology*. Section 3, Fascicle 8, p.97 Washington DC: US Armed Forces Institute of Pathology.

SALTER, M., SOLLACCIO, R.J., BERNREUTER, W.K. & WEPPELMANN, B. (1989). Primary lymphoma of bone: the use of MRI in pre-treatment evaluation. *Am. J. Clin. Oncol.*, **12**, 101.

SHOJI, H. & MILLER, T.R. (1971). Primary reticulum cell sarcoma of bone. Significance of clinical features upon the prognosis. *Cancer*, **28**, 1234.

STOKES, S.H. & WALZ, B.J. (1983). Pathologic fracture after radiation therapy for primary non-Hodgkin's malignant lymphoma of bone. *Int. J. Radiat. Oncol. Biol. Phys.*, **9**, 1153.

SUSNORWALA, S.S., DINSHAW, K.A., PANDE, S.C. & 3 others (1990). Primary lymphoma of bone: experience of 39 cases at the Tata Memorial Hospital. India. *India. J. Surg. Oncol.*, **44**, 229.

SUTCLIFFE, S.B., GOSPODAROWICZ, M.K., BUSH, R.S. & 7 others (1985). Role of radiation therapy in localized non-Hodgkin's lymphoma. *Radiother. Oncol.*, **4**, 211.

The non-Hodgkin's Lymphoma Pathologic Classification Project (1982). National Cancer Institute sponsored study of classifications of non-Hodgkin's lymphomas. Summary and description of a Working Formulation for clinical usage. *Cancer*, **49**, 2112.

WANG, C.C. & FLEISCHLI, D.J. (1968). Primary reticulum cell sarcoma of bone with emphasis on radiation therapy. *Cancer*, **22**, 1968.